# *XPO1* Gene Therapy Attenuates Cardiac Dysfunction in Rats with Chronic Induced Myocardial Infarction

**DOI:** 10.1007/s12265-019-09932-y

**Published:** 2019-11-25

**Authors:** María García-Manzanares, Estefanía Tarazón, Ana Ortega, Carolina Gil-Cayuela, Luis Martínez-Dolz, José Ramón González-Juanatey, Francisca Lago, Manuel Portolés, Esther Roselló-Lletí, Miguel Rivera

**Affiliations:** 1 Department of Animal Medicine and Surgery, Veterinary Faculty, Universidad Cardenal Herrera-CEU, CEU Universities, Valencia, Spain; 2 Myocardial Dysfunction and Cardiac Transplantation Unit, Health Research Institute Hospital La Fe (IIS La Fe), Avd. Fernando Abril Martorell, 106, 46026 Valencia, Spain; 3 Heart Failure and Transplantation Unit, Cardiology Department, University Hospital La Fe, Valencia, Spain; 4 Cellular and Molecular Cardiology Research Unit, Department of Cardiology and Institute of Biomedical Research, University Clinical Hospital, Santiago de Compostela, Spain; 5 Department of Animal Production and Health, Veterinary Public Health and Food Science and Technology, Veterinary Faculty, Universidad Cardenal Herrera-CEU, CEU Universities, Valencia, Spain

**Keywords:** Gene silencing, Myocardial infarction, Ventricular function, *XPO1*

## Abstract

Transcriptomic signature of *XPO1* was highly expressed and inversely related to left ventricular function in ischemic cardiomyopathy patients. We hypothesized that treatment with AAV9-shXPO1 attenuates left ventricular dysfunction and remodeling in a myocardial infarction rat model. We induced myocardial infarction by coronary ligation in Sprague-Dawley rats (*n* = 10), which received AAV9-shXPO1 (*n* = 5) or placebo AAV9-scramble (*n* = 5) treatment. Serial echocardiographic assessment was performed throughout the study. After myocardial infarction, AAV9-shXPO1-treated rats showed partial recovery of left ventricular fractional shortening (16.8 ± 2.8 vs 24.6 ± 4.1%, *P* < 0.05) and a maintained left ventricular dimension (6.17 ± 0.95 vs 4.70 ± 0.93 mm, *P* < 0.05), which was not observed in non-treated rats. Furthermore, lower levels of EXP-1 (*P* < 0.05) and lower collagen fibers and fibrosis in cardiac tissue were observed. However, no differences were found in the IL-6 or TNFR1 plasma levels of the myocardium of AAV9-shXPO1 rats. AAV9-shXPO1 administration attenuates cardiac dysfunction and remodeling in rats after myocardial infarction, producing the gene silencing of *XPO1*.

**Electronic supplementary material:**

The online version of this article (10.1007/s12265-019-09932-y) contains supplementary material, which is available to authorized users.

## Introduction

Coronary heart disease carries significant morbidity and is the leading cause of death across all diseases of the circulatory system [1]. After myocardial infarction, adverse ventricular remodeling associated with a higher probability of heart failure and mortality occurs [2, 3], and numerous cellular and molecular pathways are affected [4–7], such as the existence of various alterations in the molecular machinery of nuclear-cytoplasmic transport [8, 9], which precisely regulates the bidirectional selective protein flow between the nucleus and the cytoplasm.

Previous studies have shown that several molecules that participate in nuclear-cytoplasmic transport (Exportin-1 [EXP-1], IMP-β3, Nup160) are intimately related to a reduced left ventricular (LV) function in human ischemic cardiomyopathy. The transcriptomic signature of these alterations has been found and has been identified that changes in gene expression, specifically of *XPO1* that encodes EXP-1, were highly related to LV dysfunction in patients with ischemic cardiomyopathy [10].

The short hairpin RNA (shRNA) can be used to silence specific genes and is a powerful tool in studies pertaining to loss of gene function and characterization. The highly cardiotropic adeno-associated virus vector (AAV), with high affinity for the heart and down to other organs, can be introduced simply by intravenous injection [11, 12]. In particular, AAV9 has a great potential as a valuable tool for cardiac therapy in cardiovascular disease experimental models for RNA interference and gene therapy [13].

We hypothesize that manipulation of gene deregulation has therapeutic value in myocardial infarction. We aim to investigate whether highly significant relationship between *XPO1* and ventricular function is a component of causality. Therefore, we have developed a rodent myocardial infarction experimental model to show whether AAV9-shXPO1 silencing agent induces recovery of myocardial function.

## Methods

### Ethics Statement

The project was approved by the Biomedical Investigation Ethics Committee of Hospital La Fe. The investigation conforms to the *Guide for the Care and Use of Laboratory Animals* published by the US National Institutes of Health (NIH Publication No. 85-23, revised 1996), and the National (RD 53/2013) and European Directive (2010/63/EC). All surgery was performed using accurate anesthesia and analgesia veterinary protocols, to minimize animal suffering. All animal procedures are reported following ARRIVE (Animal Research: Reporting of In Vivo Experiments) guidelines and included as Supplementary Data (Online Resource 1).

### Rat Myocardial Infarction Model

**Fig. 1:**
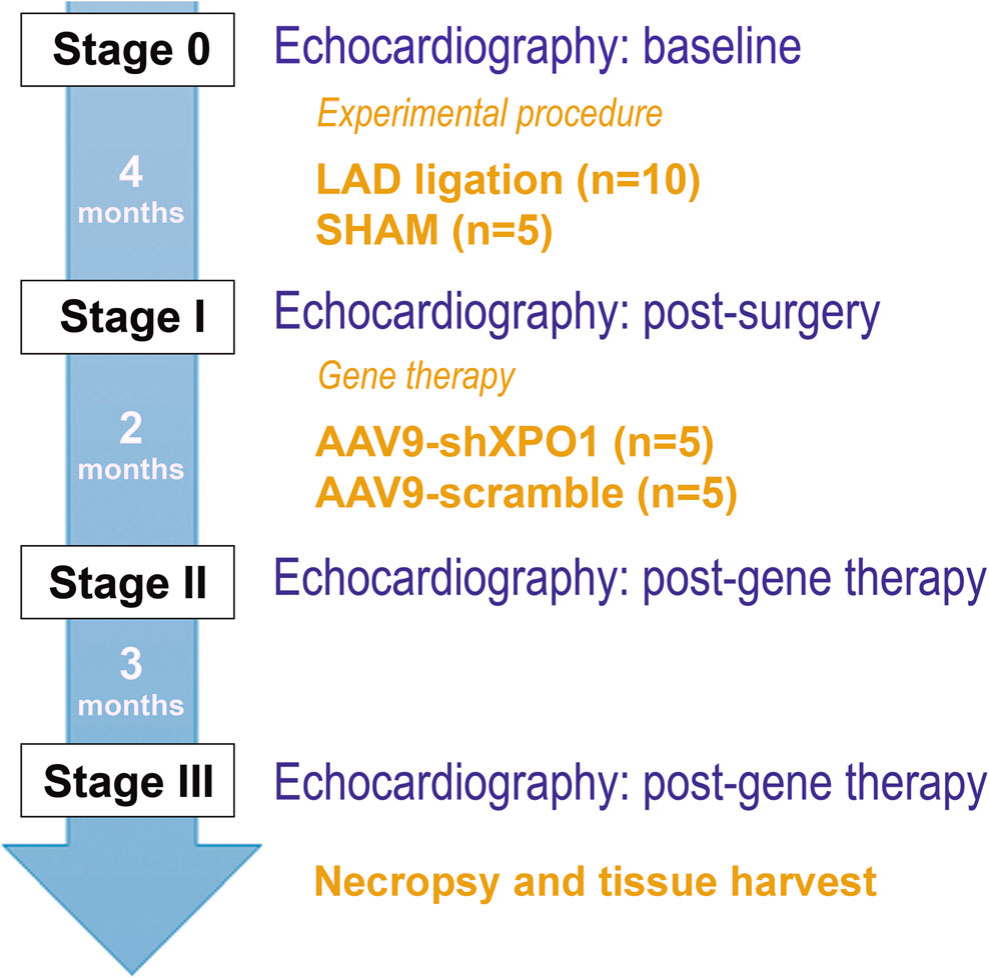
Study protocol timeline. Baseline functional echocardiographic parameters were evaluated (stage 0) in rats before the experimental procedure. Functional parameters were studied in sham and LAD (left anterior descending) coronary artery ligation animals after procedure (stage I). Four months later, the animals were administered with either adeno-associated virus vector silencing *XPO1* gene (AAV9-shXPO1) or placebo (AAV9-scramble) particles. After gene therapy, changes in functional parameters were evaluated by echocardiography 2 (stage II) and 5 (stage III) months later

Adult male Sprague-Dawley rats weighting 300–400 g were anesthetized and left anterior descending (LAD) coronary artery ligation was performed (*n* = 10) with polypropylene non-absorbable sutures (Premilene® Braun) to induce chronic myocardial infarction (Online Resource 1). Tetrazolium staining was performed in transversal slices from infarcted hearts 6 h after LAD coronary artery banding in preliminary myocardial infarction rats to ensure technical procedure [14]. Four months later, a group of infarcted rats showing left ventricular systolic dysfunction received intravenous AAV9-shXPO1 gene therapy (*n* = 5), and another received intravenous placebo AAV9-scramble (*n* = 5) (see below). Age-matched non-infarcted sham rats (*n* = 5) served as healthy non-failing rats, not receiving any treatment. The experimental design of these groups and the experimental procedure (Fig. 1) were performed based on previous studies focused on this field of research [15–17].

### Gene Therapy

AAV9-shXPO1 and placebo AAV9-scramble were manufactured by Creative Biogene (Shirley, NY). Based on previous literature, different AAV9 particles were administered (5 × 10^11^ genomes) [13] by tail vein injection at 16 weeks after infarction in infarcted rats (AAV9-shXPO1, *n* = 5; AAV9-scramble, *n* = 5). Five months after vector delivery, the rats were killed, and tissue (heart, brain, skeletal muscle, and liver) and peripheral blood samples were obtained. All samples were stored at − 80 °C until protein extraction.

### Echocardiographic Assessment

A non-invasive transthoracic echocardiographic method was used to evaluate the morphology and function of the left ventricle. Echocardiography was performed on anesthetized animals with ketamine and valium (26 and 6 mg/kg, respectively) and so their anterior chest was shaved. Heart functional parameters were analyzed with a two-dimensional mode using Philips EnVisor M2540A Ultrasound System before the LAD ligation and AAV9-shXPO1/AAV9-scramble administration as well as 2 and 5 months after gene therapy.

### Tissue Sampling

Frozen samples from AVV9-scramble (*n* = 5) and AAV9-shXPO1 (*n* = 5) groups (50 mg of heart, brain, skeletal muscle, and liver) were homogenized in a total protein extraction buffer (2% SDS, 10 mM EDTA, 6 mM Tris-HCl, pH 7.4) with protease inhibitors (25 μg/ml aprotinin and 10 μg/ml leupeptin) in a FastPrep-24 homogenizer with specifically designed Lysing Matrix D tubes (MP Biomedicals, USA). The homogenates were centrifuged and the supernatants were aliquoted. The protein content of the aliquots was determined by Peterson’s modification [18] of the Lowry method using bovine serum albumin (BSA) as standard. Cardiac samples were obtained from left ventricles, including infarcted area [19].

### Western Blot Analysis

Protein samples for detection of EXP-1 and GAPDH were separated using Bis-Tris Midi gel electrophoresis with 4–12% polyacrylamide under reducing conditions. Description of Western blot procedure is extensively described by Ortega et al. [20]. The primary detection antibodies used were anti-Exportin-1 (611833) mouse monoclonal antibody (1:50) from BD Transduction Laboratories™, and anti-GAPDH (ab9484) mouse monoclonal antibody (1:1000) obtained from Abcam and used as a loading control.

The bands were visualized using an acid phosphatase–conjugated secondary antibody and nitro blue tetrazolium/5-bromo-4-chloro-3-indolyl phosphate (NBT/BCIP, Sigma-Aldrich, St. Louis, USA) substrate system. Finally, the bands were digitalized using an image analyzer (DNR Bio-Imagining Systems, Israel) and quantified with the GelQuant Pro (v. 12.2) program. All the experiments were performed in triplicate.

### Enzyme-Linked Immunoassay and Histological Analysis

EXP-1 tissue (heart, brain, skeletal muscle, and liver) levels and IL-6 and TNFR1 plasmatic levels were determined by enzyme-linked immunosorbent assay in triplicate using the ELISA Kit for Exportin-1 (EXP-1), interleukin-6 (IL-6), and tumor necrosis factor receptor 1 (TNFR1) from Cloud-Clone Corp. (Katy, TX, USA). Additionally, Masson’s trichrome staining was performed to observe fibrotic myocardium.

### Statistical Methods

The Kolmogorov-Smirnov test was used to analyze the distribution of the variables. All variables were normally distributed. Data are presented as mean value ± standard deviation. Comparisons of variables were analyzed using two-way ANOVA and Student’s *t* test. Significance was accepted at the *P* < 0.05 level. All statistical analyses were performed using SPSS software v. 20, 2012 for Windows (IBM SPSS Inc., Chicago, IL, USA).

## Results

### *XPO1* Silencing, AAV9-shXPO1 Specificity, and Histological Analysis

**Fig. 2:**
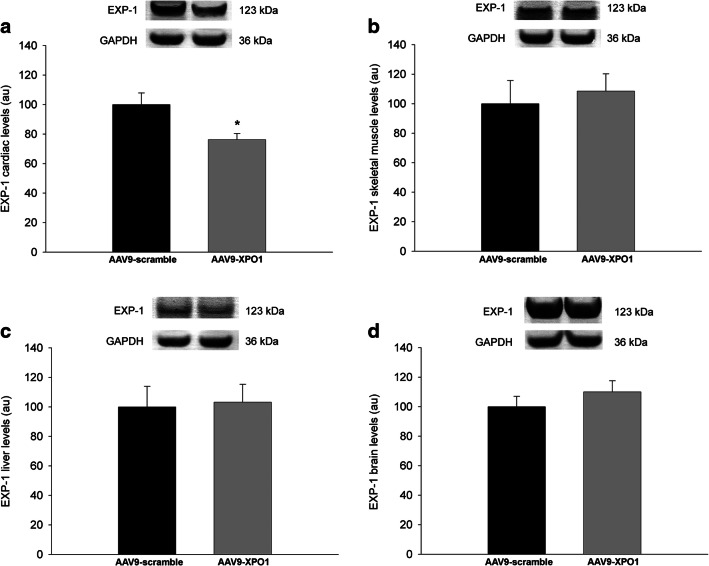
EXP-1 protein levels in AVV9-shXPO1 and AAV9-scramble rats. **a** Heart. **b** Skeletal muscle. **c** Liver. **d** Brain. The values are normalized to GAPDH, and then to the AAV9-scramble group. The values from the AAV9-scramble group were set to 100. The data are expressed as mean ± SEM in arbitrary units. Au, arbitrary units. *P < 0.05

**Fig. 3:**
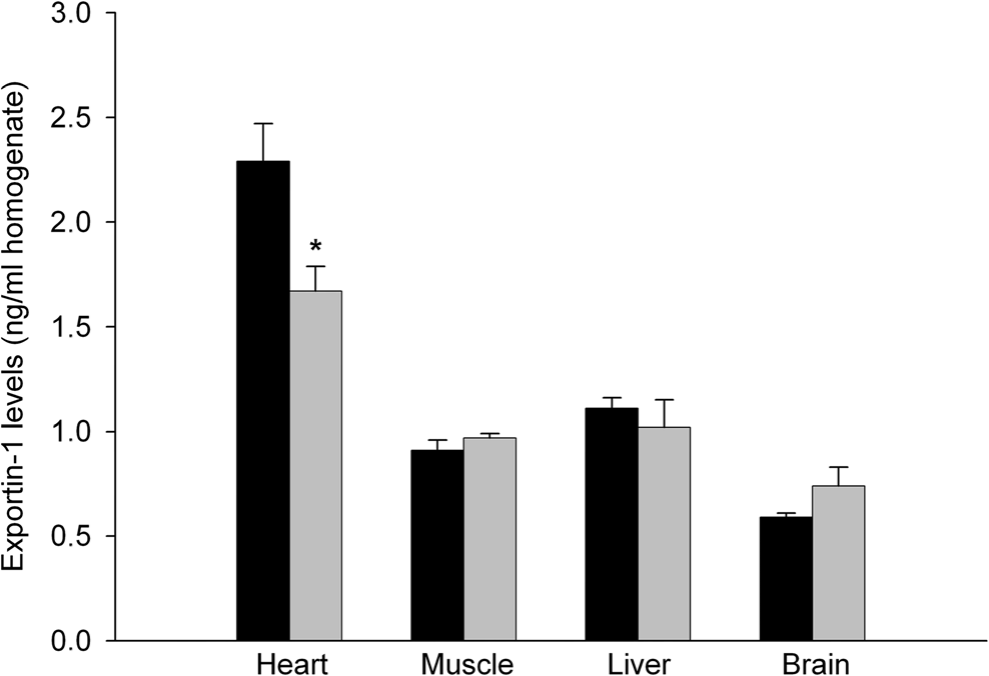
EXP-1 levels determined by immunoassay in infarcted AAV9-shXPO1 and AAV9-scrmable rats. Black bars, AVV9-scramble group; gray bars, AVV9-shXPO1 group. *P < 0.05

**Fig. 4:**
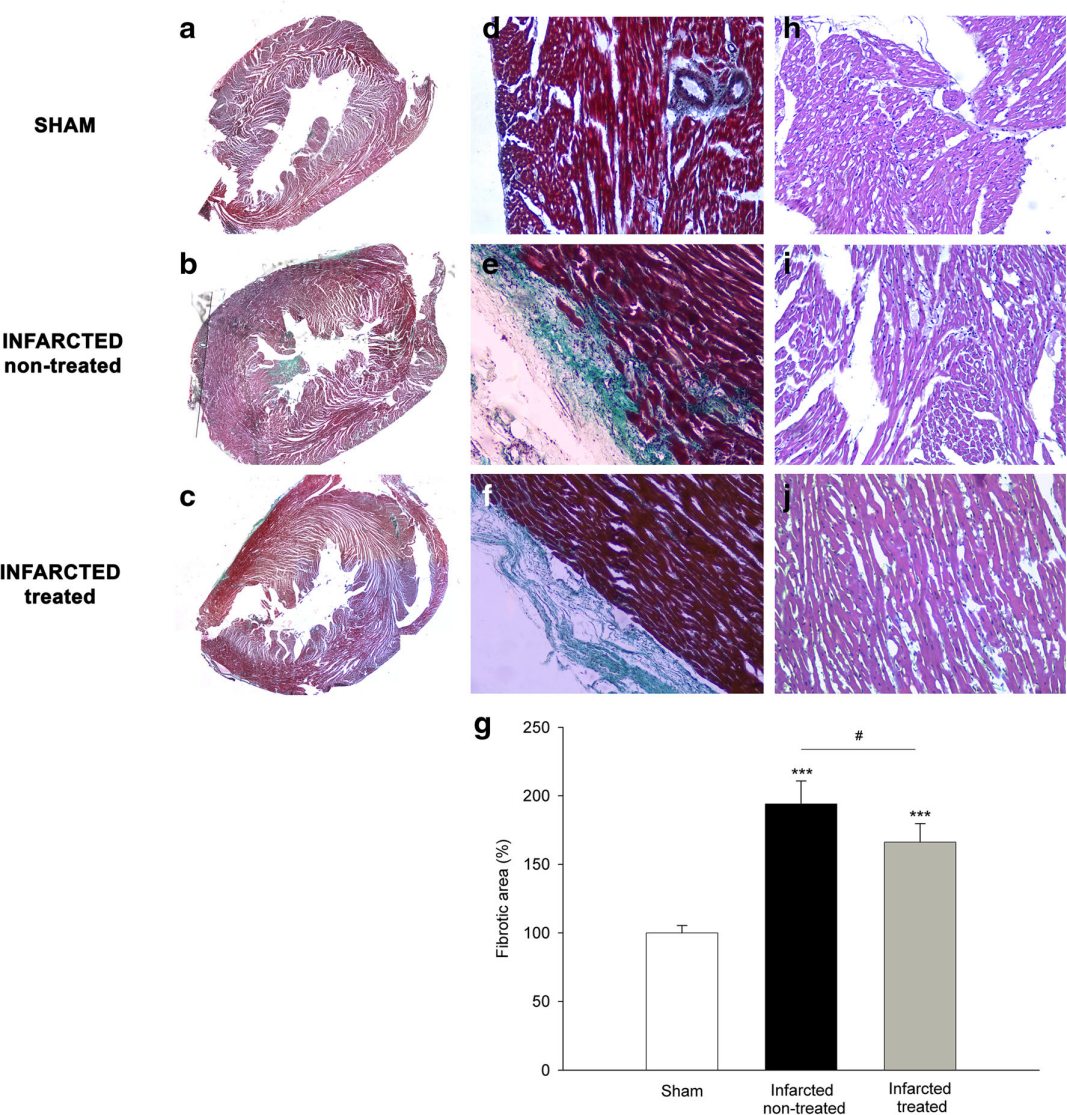
Masson’s trichrome staining in sham (**a**), AAV9-scramble (**b**), and AAV9-shXPO1 (**c**) rat hearts. Detail of the ×10 sections of sham (**d**), AAV9-scramble (**e**), and AAV9-shXPO1 (**f**) rats. **g** Percentage of fibrotic areas in all groups of rats. Hematoxylin-eosin staining of sham (**h**), AAV9-scramble (**i**), and AAV9-shXPO1 (**j**) rats. ***P < 0.001 sham vs AAV9-scramble and AAV9-shXPO1; ^#^*P* < 0.05 AAV9-scramble vs AAV9-shXPO1

To determine the efficacy of *XPO1* silencing and specificity of the AAV9-shXPO1 vector, we measured EXP-1 levels in different explanted tissues of rats by Western blot. Compared with the AVV9-scramble group, the AVV9-shXPO1 group showed lower EXP-1 levels in cardiac tissue (100 ± 16 vs 76 ± 9 arbitrary units, au, *P* < 0.05) (Fig. 2a). Both the AVV9-scramble and the AVV9-shXPO1 groups had similar EXP-1 levels in the skeletal muscle, liver, and brain (100 ± 31 vs 109 ± 26 au, 100 ± 28 vs 103 ± 25 au, and 100 ± 14 vs 110 ± 17 au, *P* > 0.05; respectively) (Fig. 2b–d). We also confirmed lower left ventricular EXP-1 expression levels by ELISA analysis in the AAV9-shXPO1 group (2.29 ± 0.18 vs 1.67 ± 0.12 ng/ml, *P* < 0.05) (Fig. 3). Additionally, IL-6 and TNFR1 plasmatic levels show no significant differences between AAV9-scramble and AAV9-shXPO1 groups (IL-6, 35.68 ± 5.47 vs 38.59 ± 7.77 pg/ml, *P* = 0.510; TNFR1, 2.74 ± 0.43 vs 2.93 ± 0.38 ng/ml, *P* = 0.470, respectively). No secondary effects produced by the AAV9-shXPO1 vector were observed. Masson’s trichrome staining shows differences in collagen fibers and fibrosis among the myocardium of AAV9-shXPO1 and AAV9-scramble rats (Fig. 4).

### Echocardiographic Assessment

Cardiac function in infarcted and sham rats was measured by non-invasive transthoracic echocardiography to evaluate ventricular function and diameter. Echocardiographic measurements were taken prior to surgery and treatment of rats (stage 0), after coronary ligation (stage I) and 2 (stage II) and 5 (stage III) months after AAV9 injection.

The rats were under strict supervision of highly qualified personnel, maintaining precise control of the anesthesia of the animal, with low intragroup variation heart rate throughout the follow-up (sham 353 ± 16, AAV9-shXPO1 367 ± 12, and AAV9-scramble 450 ± 25 beats/min).

**Fig. 5:**
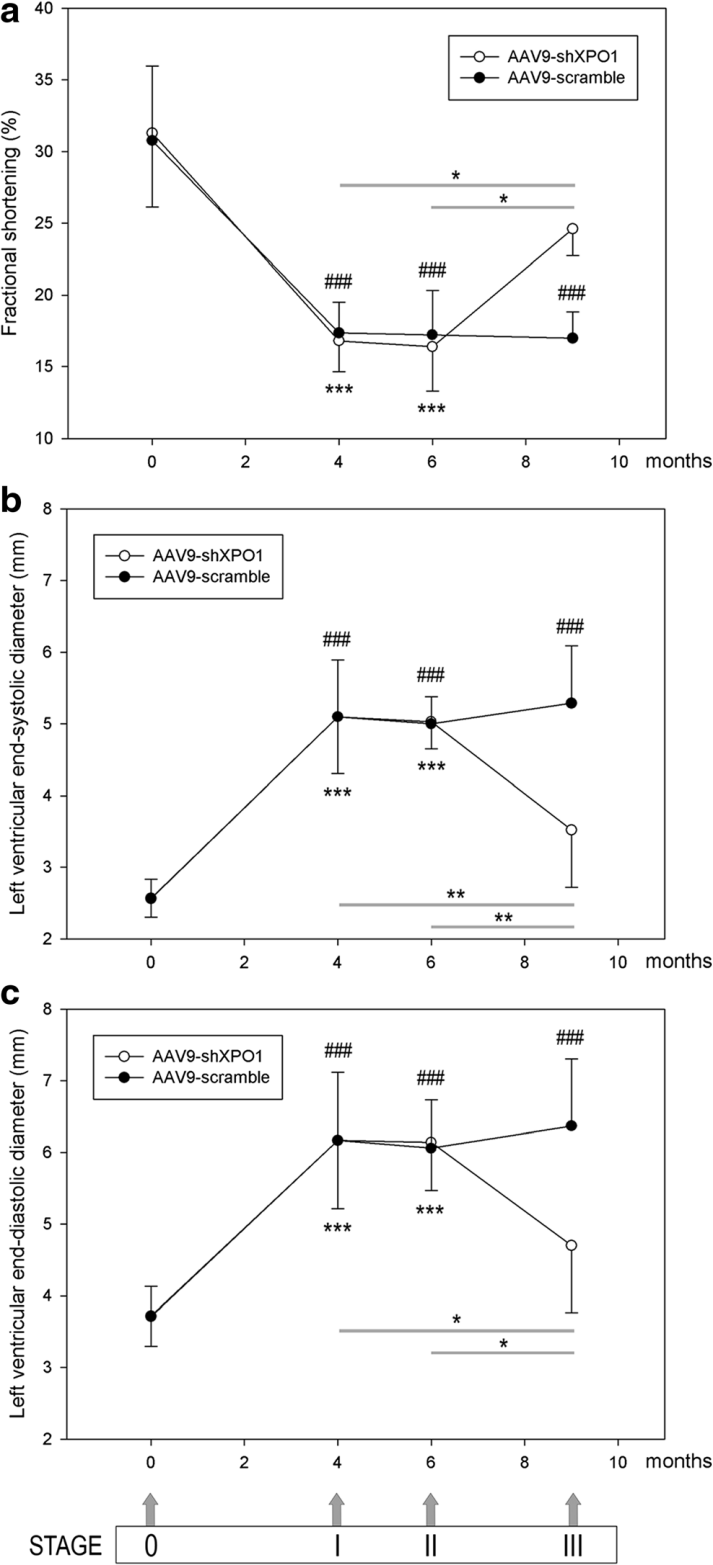
Echocardiographic parameters of infarcted AAV9-shXPO1 and AAV9-scramble rats measured before surgery and injection (stage 0), before injection (stage I), and 2 (stage II) and 5 months (stage III) after vector injection. **a** Fractional shortening. **b** Left ventricular end-systolic diameter. **c** Left ventricular end-diastolic diameter. ^###^*P* < 0.001 stage 0 vs stages 1, 2, and 3 in AAV9-scramble; ***P < 0.001 stage 0 vs stages 1 and 2 in AAV9-shXPO1; *P < 0.05 and **P < 0.01 stage 3 vs stages 1 and 2 in AAV9-shXPO1

We found differences in echocardiographic parameters depending on the stage but not regarding study groups. As shown in Fig. 5, fractional shortening (FS) of the AAV9-shXPO1 infarcted rat group was 31.3 ± 8.6% at stage 0, 16.8 ± 2.8% at stage I, 16.4 ± 2.4% at stage II, and 24.6 ± 4.1% at stage III (*P* < 0.05 compared with stage I, Fig. 5a). The AAV9-scramble rat group had a FS of 30.8 ± 7.6% at stage 0, 16.7 ± 2.7% at stage I, 16.5 ± 2.5% at stage II, and 16.5 ± 2.6% at stage III. Sham rats had normal FS (31.1 ± 8.0%, stage 0) that is maintained throughout the study.

LV end-systolic (LVESD) and LV end-diastolic diameters (LVEDD) of AAV9-shXPO1 rats were 2.57 ± 0.60 mm and 3.73 ± 0.63 mm, at stage 0, 5.10 ± 0.79 mm and 6.17 ± 0.95 mm at stage I, 5.03 ± 0.87 mm and 6.14 ± 1.14 mm at stage II, and 3.52 ± 0.88 mm and 4.70 ± 0.93 mm at stage III (*P* < 0.01 and *P* < 0.05 compared with stage I, Fig. 5b, c), respectively. Sham rats had normal LVESD (2.53 ± 0.61 mm) and LVEDD (3.75 ± 0.59 mm) that are maintained throughout the study.

## Discussion

This study may provide a new therapeutic strategy based on gene therapy to restore ventricular function in patients with ischemic cardiomyopathy, which is the leading cause of death worldwide and lacking effective treatment [21]. In this study, we report a successful delivery of an AAV-based gene therapy in a long-term chronic myocardial infarction rat model, which simulates the clinical features seen in patients with coronary heart disease after myocardial infarction. We have expanded the follow-up until 5 months after gene delivery, proving the long-term efficacy of the treatment and showing the safety of this procedure, since there was no evidence of side-effects in the animals.

EXP-1 mediates the nuclear export of proteins, rRNA, snRNA, and some mRNAs. Previous studies in patients with ischemic cardiomyopathy showed elevated expression levels of both EXP-1 mRNA and protein, and interestingly, these levels were inversely related with ejection fraction and positively correlated with LVESD and LVEDD [8, 9], i.e., higher EXP-1 expression is linked with LV function impairment. Hence, we intended to demonstrate the cause-effect of this relationship in this study through gene therapy, which may result in a useful approach for the treatment of heart disease [22–24]. Cardiac gene therapy uses vectors that can robustly, specifically, and persistently deliver therapeutic genetic materials to the heart without generating local and/or systemic toxicity. The adenoviral vectors represent an efficient but unstable gene delivery vector for the heart. Nonetheless, long-term myocardial transduction in adult animals has been accomplished with the development of AAV [25]. Research has established AAV9 as a cardiotropic vector superior to all the other serotypes in rodents, making it the most appropriate vector for gene delivery to the heart [11, 12]. Our results support this property of AAV9, since we observed that the cardiac tissue was the only sample analyzed where EXP-1 levels decreased in infarcted AAV9-shXPO1 rats compared with those in AAV9-scramble rats. Furthermore, we show the effectiveness and stability of the vector AAV9-shXPO1 as EXP-1 levels decreased in heart tissue 5 months after transduction. Regarding the magnitude of the effect, systematic or local, our results do not show differences in the inflammatory parameters studied in rats treated with AAV9-shXPO or AVV9-scramble.

LV function parameters are directly related to ventricular remodeling that occurs after injury of the heart muscle. Ventricular function of infarcted rats appeared to be in partial recovery following *XPO1* silencing. At 2 months after injection (stage II), cardiac function in the rats was similar to that immediately after coronary ligation (stage I). Nevertheless, at 5 months after injection, the differences in these parameters were significant and resulted in improvements in the state of cardiac function in infarcted rats with *XPO1* silencing. Furthermore, we have observed a decrease in fibrosis after treatment. This silencing could have similar effects at revascularization, recovering the hibernating myocardium and thereby improving ventricular function in infarcted rats. Although this partial recovery would be slower than that achieved by performing a bypass procedure, it is less invasive and harmful. We did not observe an improvement in the cardiac function of AAV9-scramble infarcted rats; these rats maintained similar parameters during the follow-up after coronary ligation.

## Study Limitations

In order to rationalize funding resources and minimize animal testing, we decided to compare only gene therapy responses in rats with chronic infarction, not studying AAV9-shXPO1 administration in healthy control rats [11]. For the development of the myocardial infarction model, a standardized protocol of veterinary pharmacology and surgery was followed; still, the manual procedure of coronary ligation may introduce some variability between infarcted rats.

## Conclusions

In conclusion, AAV9-shXPO1 administration attenuates cardiac dysfunction in rats after myocardial infarction, producing the gene silencing of XPO1. This study provides a new therapeutic strategy based on gene therapy to restore ventricular function in patients with ischemic cardiomyopathy.

## Clinical Relevance

This study offers a new way to restore cardiac function in patients who have suffered from myocardial infarction, by gene therapy through the silencing of *XPO1.*

## Electronic Supplementary Material

ESM 1 (DOCX 221 kb)

## Abbreviations

AAV: Adeno-associated virus vector
EXP-1: Exportin-1
FS: Fractional shortening
IL-6: Interleukin-6
LAD: Left anterior descending
LV: Left ventricular
LVEDD: Left ventricular end-diastolic diameter
LVESD: Left ventricular end-systolic diameter
TNFR1: Tumor necrosis factor receptor 1
*XPO1*: Exportin-1 gene
