# Supplementary material for: XPO1 Gene Therapy Attenuates Cardiac Dysfunction in Rats with Chronic Induced Myocardial Infarction
Source: J Cardiovasc Transl Res. 2019 Nov 25;13(4):593–600. doi: 10.1007/s12265-019-09932-y (PMC7423868; doi:10.1007/s12265-019-09932-y)
Supplement: Supplementary file 1 — (DOCX 221 kb) [file 12265_2019_9932_MOESM1_ESM.docx]

***XPO1* gene therapy attenuates cardiac dysfunction in rats with chronic induced myocardial infarction**

**María García-Manzanares^1,2¶^, Estefanía Tarazón^2^¶^, Ana Ortega^2^, Carolina Gil-Cayuela^2^^, Luis Martínez-Dolz^2,3^^, José Ramón González-Juanatey^4^^, Francisca Lago^4^^, Manuel Portolés^2#^*^, Esther Roselló-Lletí^2#^^ and Miguel Rivera^2#^^**

**Protocol. Experimental procedure in rats**

**Myocardial infarction**

**Anesthesia and analgesia**

To perform the infarction in the animals, a standardized anesthetic protocol was used. The first step consisted in the induction of the animal kept in an anesthetic chamber with a mixture of oxygen and sevoflurane (5% v/v Abbott) for approximately 2 minutes or until a cessation of motor activity was observed. Endotracheal intubation was carried out with an 18 G (1.3 mm diameter) catheter and a 2 "(5.1 cm) length (Braun). For this, the rat was placed in the supine position on a platform and the limbs were clamped to stabilize the position. By lighting with a cold light source directed to the ventral area of the neck and gently extracting the tongue, the glottis and trachea of the rat were visualized. Intubation was performed and the rat was immediately connected to an automatic respirator (Harvard Apparatus^®^, model 683) with set parameters of 2 ml of tidal volume and 140 breaths per minute. The correct intubation was checked by confirming breath on a glass surface first and ensuring rhythmic movement of the chest with respect to assisted ventilation afterwards. The tube was fixed to the animal and to the surgical table. The appropriate anesthetic level was maintained during the surgical procedure with 2% sevofluorane, 0.3 ml of N_2_O and 0.1 ml of O_2_. The rat was placed on a cork surface and covered with a surgical drape. An analgesic drug, fentanyl (Fentanest^®^, Kern Pharma) was used at a dose of 0.05 mg/kg inoculated immediately before starting surgery intraperitoneally (IP) as well as a dose of 0,05 mg/kg IP of buprenorphine (Buprex^®^, Schering-Plow SA) 20 minutes before procedure. In order to avoid muscle contractions, 6 mg/kg lidocaine dose was infiltrated intraincisionally at the beginning of the procedure. In the post-surgical protocol, animals were given a dose of 0.05 mg/kg of buprenorphine IP every 6-8 hours for 48 hours. The euthanasia of the rats was carried out according to the regulations in force, administering an anesthetic dose of ketamine/diazepam and performing a subsequent exsanguination, or inoculating 1 ml of intracardiac 0.2 M KCl.

**Surgical procedure**

The rat was placed in the supine position on an isolated cork base and the limbs were immobilized with adhesive tapes. The eyes were hydrated with a gel tear to avoid corneal ulcers (Lubrifilm^®^, Alcon Cusí). For the preparation of the surgical area, the thorax was shaved and disinfected with diluted povidone (Betadine^®^, Viatris Manufacturing). Animals were covered with a surgical drape leaving the thorax free (Fig 1) to perform a lateral thoracotomy between the 4th and 5th left intercostal space.


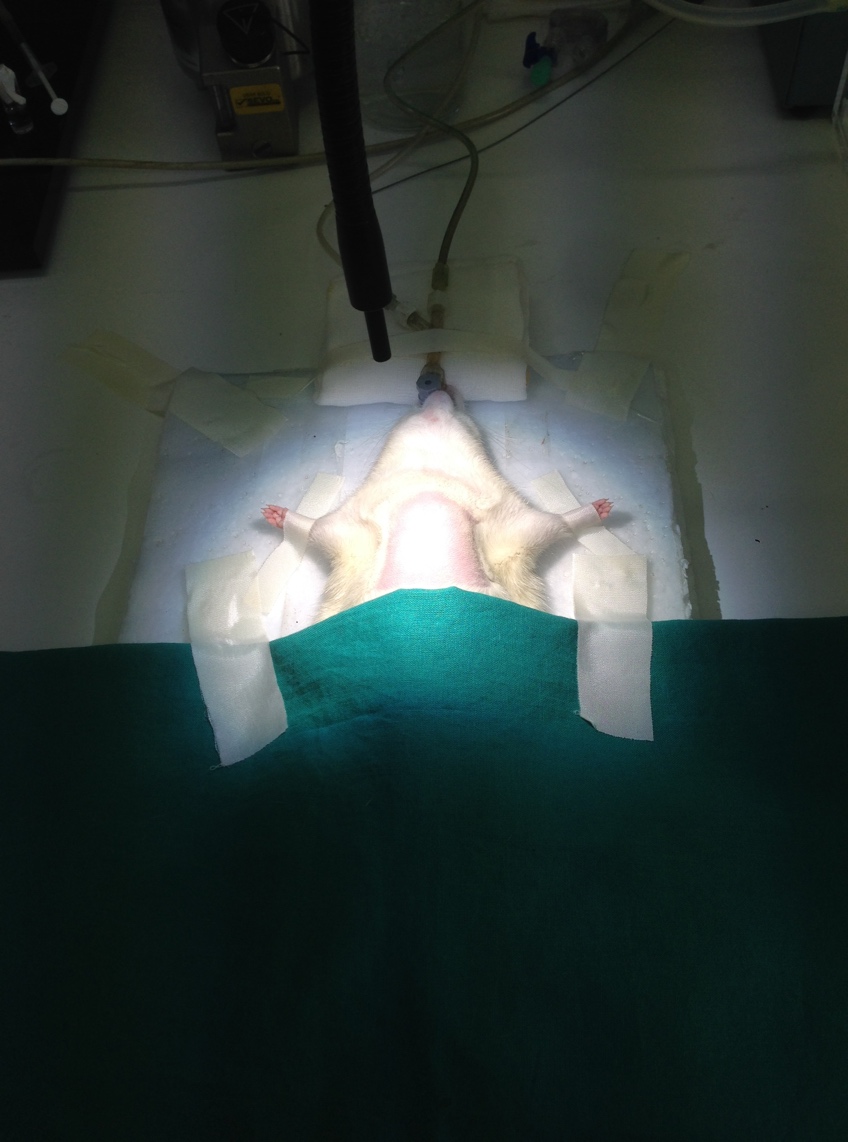


**Fig 1. Procedure preparation of animals.**

First, a two-centimeter skin incision was made directed towards the sternum and middle area of ​​the thorax. The subcutaneous tissue was separated and after the dissection of the pectoral and intercostal muscles (the latter cranially to the rib to avoid the vascular bundle that runs caudally to them) was accessed to the thoracic cavity. By placing spacers in the intercostal space and gauze below the thorax of the rat it was possible to expose the heart. Then, with the help of a swab soaked in physiological saline, the thymus was removed cranially. Then, with gentleness and avoiding the cranial lung lobe, pericardiotomy was performed incising with blunt microsurgical forceps. The area was cleaned with a wet and sterile gauze to be able to visualize the left anterior descending coronary artery and ligation area was delimitated starting from about 4 mm below the left atrium. For the occlusion, 5/0 non-absorbable monofilament suture (Premilene^®^, Braun) was used by permanent ligation. Correct ligation of the artery and infarction was evidenced, due to the change that occurs immediately in the infarcted area (bluish color).

Finally, the thorax was sutured with a 3/0 resorbable monofilament suture (Monosyn^®^, Braun), ensuring that there was no thoracic permeability so that negative chest pressure recovered after closure. The muscular layer was closed with a continuous suture and the tissues were irrigated with saline. Finally, the skin was closed with an inthermal suture and the wound was disinfected with povidone. A few minutes after removing the inhalation agent they began to see respiratory reflexes and ventilations of the animal, at which point it was disconnected and left isolated and tempered in a cage with an electric blanket.

**Echocardiographic assessment**

To evaluate the ventricular function of the rats, previous and postoperative echocardiograms were performed. In this way, detailed information on the cardiac morphology of each individual and its evolution over time was obtained. A combination of anesthetic drugs was administered for the immobilization of the animals and to be able to take the images. A dose of 26 mg/kg of ketamine (Ketalar^®^, Pfizer) and 6 mg/kg of diazepam (Valium^®^, Roche) was prepared and 15 minutes prior to echocardiography half of the dose was inoculated to the rat, inoculating the remaining one if was necessary. The thorax was shaved and the animal was placed on an electric blanket in supine position. For the acquisition of images, the Philips EnVisor M2540A Ultrasound System was used together with a linear 10 MHz transducer. Imaging was performed 1 to 3 days before surgery, to record the baseline ventricular function of the rats, post-infarction was performed 1 week, and 2 and 5 months after gene therapy. In the study, transthoracic echocardiography was performed on the short axis with 3 cuts: one behind the atria, another cut in the middle ventricular area at the level of the papillary muscles and finally another in the apex area. Images were taken in M-mode (linear image to which the time variable has been introduced) and also in 2D mode (two-dimensional image in real time). To calculate the functional parameters, three consecutive cardiac cycles were measured using standard methods. The diameters of the left ventricle were measured in systole and diastole in the M-mode and the internal area in the 2D mode. From these measurements, the shortening fraction and the ejection fraction were calculated. In the selection of AVV9-shXPO1 and AVV9-scramble infarcted groups, we tried to include homogeneous fractional shortening among both groups and distribute them randomly.
